# Health Insurance and Interhospital Transfer for Critically Ill Patients With Respiratory Failure

**DOI:** 10.1001/jamanetworkopen.2025.28889

**Published:** 2025-08-26

**Authors:** Emily A. Harlan, Muhammad Ghous, Noella Cortinas, Nandita R. Nadig, Kelly C. Vranas, Mari Armstrong-Hough, Sarah L. Krein, Thomas S. Valley

**Affiliations:** 1 Division of Pulmonary and Critical Care, Department of Medicine, University of Michigan, Ann Arbor; 2 Center for Bioethics and Social Sciences in Medicine, University of Michigan Medical School, Ann Arbor; 3 Institute for Healthcare Policy and Innovation, University of Michigan, Ann Arbor; 4 Center for Clinical Management Research, VA Ann Arbor Healthcare System, Ann Arbor, Michigan; 5 Division of Pulmonary and Critical Care Medicine, Department of Medicine, Northwestern University, Chicago, Illinois; 6 Division of Pulmonary, Allergy, and Critical Care, Oregon Health & Science University School of Medicine, Portland; 7 Departments of Social & Behavioral Sciences and Epidemiology, New York University School of Global Public Health, New York; 8 Division of General Medicine, Department of Medicine, University of Michigan, Ann Arbor

## Abstract

**Importance:**

Critically ill patients with acute respiratory failure may benefit from transfer to higher-volume centers with specialized care. However, health insurance is often considered prior to interhospital transfer and may represent a factor other than severity of illness that influences transfer processes and outcomes for patients with respiratory failure.

**Objective:**

To examine the association between patient health insurance, interhospital transfer, and mortality for critically ill patients with acute respiratory failure.

**Design, Setting, and Participants:**

This retrospective cohort study using data from the Premier Healthcare Database included critically ill patients aged 18 years or older with acute respiratory failure receiving mechanical ventilation and intensive care within the first 3 days of hospitalization from January 1, 2017, until September 30, 2021, at academic and community hospitals across the US. Data were analyzed from October 2023 through August 2024.

**Exposure:**

Patient health insurance, categorized as commercial, Medicaid, Medicare, uninsured, or other.

**Main Outcomes and Measures:**

Multivariable logistic regression was used to estimate the associations between patient insurance type, receipt of interhospital transfer, and mortality, accounting for age, sex, severity of illness, comorbidities, and year. A shared frailty model was used to examine timing of interhospital transfer by insurance status.

**Results:**

There were 703 392 hospital admissions of critically ill patients with acute respiratory failure receiving invasive mechanical ventilation at 824 hospitals. The mean (SD) age patient age was 60.5 (17.0) years, and 400 126 (56.89%) were male; 30 613 (4.35%) underwent interhospital transfer, and 263 261 (37.43%) died or were discharged to hospice. In adjusted analyses, there were lower odds of undergoing interhospital transfer for patients without insurance compared with commercial insurance (adjusted odds ratio [AOR], 0.56; 95% CI, 0.51-0.61; absolute difference in estimated probability of transfer, 2.39 percentage points; *P* < .001). Patients without insurance experienced significantly higher odds of mortality (AOR, 1.31; 95% CI, 1.25-1.37; *P* < .001), and having no insurance was associated with slower time to interhospital transfer (adjusted hazard ratio, 0.72; 95% CI, 0.68-0.76; *P* < .001) compared with having commercial insurance.

**Conclusions and Relevance:**

In this cohort study, a lack of health insurance was associated with lower odds of interhospital transfer and higher odds of mortality among critically ill patients. The findings suggest a need to better understand drivers of the interhospital transfer process and ensure that decisions for transfer are made equitably to improve outcomes for critically ill patients.

## Key Points

**Question:**

Is health insurance status associated with interhospital transfer and mortality for critically ill patients with acute respiratory failure in the US?

**Findings:**

In this cohort study including 703 392 hospital admissions of critically ill adults with acute respiratory failure, patients without health insurance experienced lower odds of interhospital transfer and higher odds of mortality compared with patients with commercial insurance.

**Meaning:**

The findings suggest health insurance may play a role in the interhospital transfer decision-making process, underscoring the need to evaluate hospital transfer practices to ensure equitable care delivery.

## Teaser

This cohort study examines whether patient health insurance status is associated with interhospital transfer and mortality in a nationwide population of critically ill patients with acute respiratory failure in the US.

## Introduction

Critically ill patients with acute respiratory failure may benefit from specialized care in hospitals with a high volume of patients.^1,2,3^ Care in a higher-volume center is associated with lower risk of mortality for patients receiving mechanical ventilation, possibly due to the implementation of protocolized care in the intensive care unit (ICU) or increased clinician experience managing critically ill patients.^1^ One suggested strategy to improve outcomes for critically ill patients is to systematically transfer the highest acuity patients to specialized, high-volume centers where patients may have improved outcomes.^4^ Yet, prior work has shown that these transfers do not always occur.^5,6^ The complex array of patient, clinician, and hospital factors impacting the interhospital transfer process for critically ill patients is not completely understood.^5,7,8,9^

Ideally, a patient’s health insurance should not impact health care processes or outcomes. Yet, being uninsured has been associated with reduced odds of interhospital transfer for patients with acute coronary syndrome, earlier withdrawal of life-sustaining therapy among critically ill trauma patients and patients with traumatic brain injury or spinal cord injury, and increased mortality among critically ill patients.^10,11,12,13,14,15^ While the Emergency Medical Treatment and Labor Act (EMTALA) was specifically created to ensure emergency care is provided to patients regardless of their ability to pay and requires hospitals to provide stabilizing care to all patients seen in the emergency department, EMTALA regulations do not protect patients after they are considered medically stabilized, which has typically been interpreted as after admission to the hospital.^16^ After medical stabilization, if interhospital transfer is requested, the accepting hospital is permitted to request information about a patient’s insurance status.

Therefore, we sought to examine the associations between patient health insurance, interhospital transfer, and mortality for critically ill patients admitted with acute respiratory failure and receiving invasive mechanical ventilation. We hypothesized that patients without insurance would have lower odds of interhospital transfer and higher mortality than patients with commercial insurance, Medicare, or Medicaid.

## Methods

### Patient Cohort

We conducted a retrospective cohort study using the Premier Healthcare Database (PHD). PHD is an administrative database that includes patients regardless of their health insurance status from 1271 geographically diverse hospitals in the US and contains approximately 25% of all inpatient hospitalizations in the US.^17^ We identified all adult patients (aged 18 years or older) hospitalized from January 1, 2017, until September 30, 2021, with acute respiratory failure and receiving invasive mechanical ventilation in an ICU.^17^ The study design was approved by the social, behavioral, and educational research institutional review board of the Biomedical Research Alliance of New York, which waived informed consent because patient data were deidentified. We followed the Strengthening the Reporting of Observational Studies in Epidemiology (STROBE) reporting guideline.^18^

Patients with acute respiratory failure receiving invasive mechanical ventilation were identified using *International Statistical Classification of Diseases and Related Health Problems, 10th Revision* (*ICD-10*) discharge diagnosis and procedure codes (eTable 1 in Supplement 1). We identified patients admitted to an ICU through the presence of inpatient ICU room and board charge codes.^19^ We included all ICU types (medical, coronary, surgical, trauma, cardiovascular, and burn ICUs). Neurology and intermediate care units were excluded given heterogeneity in level of care provided within these units.^20^ To reduce heterogeneity and identify patients with high severity of illness, the cohort was restricted to patients receiving early mechanical ventilation and intensive care, defined by the presence of a mechanical ventilation procedure code and an ICU room and board charge within the first 3 days of hospitalization.^7^ For patients with multiple ICU room and board charges during the first 3 days of admission, admissions were categorized according to the ICU type with higher charges. Admissions to hospitals with fewer than 5 admissions during the time frame of the study were excluded, and patients with missing sex data were excluded.

### Exposure

The exposure variable was patient health insurance, categorized as commercial, Medicaid, Medicare, uninsured (including charity, indigent, and self-pay), and other (including workers’ compensation, direct employer contracts, US Department of Defense military benefits, Indian Health Services, prison contracts, and Veterans Health Administration). Patients with Medicare or Medicaid managed care plans were included in the Medicare or Medicaid categories, respectively.

### Outcomes

The primary outcomes were interhospital transfer (ie, transfer out of 1 acute care hospital to another acute care hospital for ongoing inpatient care) and mortality. Interhospital transfers were identified using discharge status codes; in-hospital deaths were categorized as nontransferred patients. Mortality was measured using a composite of in-hospital mortality (during index hospitalization) and discharge to hospice given recognized disparities in access to hospice services and hospital variation in hospice discharge practices.^21,22,23^ This mortality measure has a high concordance with 30-day mortality.^24^ Mortality could not be assessed for patients who were discharged as interhospital transfers.

### Statistical Analysis

Multivariable logistic regression was used to estimate the association between patient health insurance status and the primary outcomes of interest (interhospital transfer and mortality). To account for patient characteristics that may confound the association between health insurance status and interhospital transfer or mortality, we adjusted analyses for patient age, sex, chronic comorbidities, severity of illness, and year of admission. Comorbidities were measured using Elixhauser *ICD-10-Clinical Modification* comorbidity codes.^25^ Severity of illness was quantified by the number of organ failures present on admission using the method previously outlined by Bosch et al^26^ and Dombrovskiy et al.^27^ Each organ failure was treated as an individual covariate; respiratory failure was excluded given that the cohort consisted of patients with respiratory failure. We computed cluster robust SE estimates to account for hospital-level clustering of patients. Adjusted odds ratios (AORs) were computed with 95% CIs. The adjusted probabilities of interhospital transfer and mortality were estimated by averaging estimated outcomes over the observed distribution of covariates using the margins command in Stata (StataCorp LLC). Analyses were performed using Stata, version 17.^28^ Two-sided *P* values <.05 were considered significant. Data were analyzed from October 2023 through August 2024.

Given that patients who die early in their hospital course may not be eligible for transfer and patients with different types of insurance may be more or less likely to die in the hospital, several analyses were conducted to account for censoring due to death when evaluating interhospital transfer. We performed a subanalysis excluding patients who died within the first 7 days of admission (ie, individuals who may not have had an opportunity for interhospital transfer). To examine the association between patient insurance status and time to interhospital transfer from hospital admission, we used a shared frailty time-to-event model with random effects, a Weibull distribution, and clustering by hospital. To assess for the competing risk of death (or discharge to hospice) in the time-to-event model, we computed a subdistribution hazard model (Fine-Gray). Adjusted hazard ratios (AHRs) and subhazard ratios were computed with 95% CIs.

To evaluate whether patients without insurance may be more likely to receive care at larger hospitals with more specialty resources and thus be less likely to require transfer, we repeated the primary analysis after excluding patients admitted to hospitals that transferred no patients during the study time frame. We additionally stratified our analysis by hospital size and volume of patients receiving mechanical ventilation given that larger hospitals may be more likely to have specialty resources and less likely to transfer patients.

During the COVID-19 pandemic, changes were made to federal reimbursement policies to better compensate hospitals for providing care to patients without insurance.^29,30,31^ To examine the implications of the COVID-19 pandemic for transfer practices, we repeated the analysis before and after the beginning of the COVID-19 pandemic. First, we examined admissions from January 2017 until March 2020, and second, we examined admissions from March 2020 until September 2021.

We performed additional subanalyses examining effect modification by severity of illness and race or ethnicity. Race and ethnicity data, as provided within the PHD, included the following categories: Asian, Black, White, Hispanic, other (not further specified in the database), and unknown. To explore differences in interhospital transfer based on federal insurance (Medicaid or Medicare) vs uninsured status, we repeated the primary analysis using patients without insurance as the reference group. We performed an analysis excluding patients admitted as interhospital transfers, as they may be less likely to be transferred again. In addition, we assessed for censoring due to transfer when evaluating mortality as an outcome.

## Results

We identified 703 392 admissions of critically ill patients with acute respiratory failure receiving invasive mechanical ventilation between 2017 and 2021 (eFigure in Supplement 1). The mean (SD) age was 60.5 (17.0) years; 303 266 patients (43.11%) were female and 400 126 (56.89%) were male. A total of 15 432 patients (2.19%) were Asian, 115 384 (16.40%) were Black, 487 832 (69.35%) were White, 62 262 (8.85%) were other race, and 22 482 (3.20%) had unknown race; 59 003 (8.39%) were Hispanic and 644 389 (91.61%) were non-Hispanic. Patients were hospitalized across 824 US hospitals. Among included hospitals, one-third (272 [33.01%]) were teaching hospitals, and 812 (98.54%) transferred at least 1 patient. Based on US Census definitions of rurality, one-fifth of included hospitals (187 [22.69%]) were in rural areas.^17^ The geographic distribution included hospitals throughout the US (Midwest, 205 [24.88%]; Northeast, 128 [15.53%]; South, 339 [41.14%]; and West, 152 [18.45%]). Over half of patient admissions (373 879 [53.15%]) were insured through Medicare, 136 285 (19.38%) through Medicaid, and 124 968 (17.77%) through commercial insurance; 42 226 (6.00%) were uninsured and 26 034 (3.70%) had other types of insurance.

Of these admissions, 30 613 (4.35%) underwent interhospital transfer and 672 779 (95.65%) did not (Table 1); 263 261 of 703 392 patients (37.43%) died or were discharged to hospice. Insurance type was similar in hospitals with fewer transfers compared with hospitals with more transfers (eTables 2 and 3 in Supplement 1). Most patient admissions (595 982 [84.73%]) were in general or medical ICUs. The most frequent principal discharge diagnoses were infectious (216 874 [30.83%]), respiratory (140 744 [20.01%]), neurologic (104 021 [14.79%]), and cardiac (90 810 [12.91%]). In the study cohort, 46 044 patients (6.55%) received tracheostomy and 80 484 (11.44%) received kidney replacement therapy during their hospitalization.

**Table 1.** Characteristics of Critically Ill Patients With Acute Respiratory Failure Receiving Invasive Mechanical Ventilation Within the First 3 Days of Hospitalization, 2017-2021, by Interhospital Transfer Status

Characteristic	Patient admissions (N = 703 392)^a^	
	Transferred	Not transferred
Total admissions	30 613 (4.35)	672 779 (95.65)
Age, mean (SD), y	56.82 (16.56)	60.68 (17.04)
Sex		
Female	12 476 (40.75)	290 790 (43.22)
Male	18 137 (59.25)	381 989 (56.78)
Race		
Asian	764 (2.50)	14 668 (2.18)
Black	4344 (14.19)	111 040 (16.50)
White	21 716 (70.94)	466 116 (69.28)
Other^b^	2760 (9.02)	59 502 (8.84)
Unknown	1029 (3.36)	21 453 (3.19)
Ethnicity		
Hispanic	2254 (7.36)	56 749 (8.44)
Non-Hispanic	28 359 (92.64)	616 030 (91.56)
Insurance		
Commercial	8013 (26.18)	116 955 (17.38)
Medicare	13 185 (43.07)	360 694 (53.61)
Medicaid	6185 (20.20)	130 100 (19.34)
Uninsured	1657 (5.41)	40 569 (6.03)
Other^c^	1573 (5.14)	24 461 (3.64)
ICU type		
General or medical	26 714 (87.26)	569 268 (84.61)
Coronary or cardiovascular	2620 (8.56)	60 194 (8.95)
Surgical or trauma	1253 (4.09)	42 230 (6.28)
Other	26 (0.08)	1087 (0.16)
COVID-19 diagnosis	1667 (5.45)	36 732 (5.46)
Principal diagnosis		
Cardiac	5071 (16.56)	85 739 (12.74)
Respiratory	6355 (20.76)	134 389 (19.98)
Gastrointestinal	1582 (5.17)	29 391 (4.37)
Infectious	9899 (32.34)	206 975 (30.76)
Neurologic	3570 (11.66)	100 451 (14.93)
Trauma or musculoskeletal	908 (2.97)	24 073 (3.58)
Hematologic or oncologic	147 (0.48)	2923 (0.43)
Kidney	242 (0.79)	6619 (0.98)
Other	2839 (9.27)	82 219 (12.22)
Organ failures at admission, mean (SD), No.^d^	1.99 (1.20)	2.05 (1.26)
Comorbidities, mean (SD), No.	5.18 (2.48)	5.64 (2.52)
Procedures		
Tracheostomy	2012 (6.57)	44 032 (6.54)
Kidney replacement therapy^e^	2942 (9.61)	77 542 (11.53)
Length of stay, median (IQR), d	4 (1-9)	8 (3-14)

Abbreviation: ICU, intensive care unit.

^a^ Data are presented as number (percentage) of admitted patients unless otherwise specified.

^b^ Other race was not further specified in the Premier Healthcare Database.

^c^ Other insurance included workers’ compensation, direct employer contracts, US Department of Defense military benefits, Indian Health Services, prison contracts, and Veterans Health Administration.

^d^ Used to assess severity of illness.

^e^ Including intermittent dialysis and continuous kidney replacement therapy.

Patients who were transferred were younger (mean [SD] age, 56.82 [16.56] years) compared with those not transferred (60.68 [17.04] years) and were more likely to have commercial insurance (8013 [26.18%] vs 116 955 [17.38%]). Transferred and nontransferred patients had similar severity of illness and chronic comorbidities. Among patients transferred, median time to transfer was 4 days (IQR, 1-9 days). Median length of hospital stay for nontransferred patients was 8 days (IQR, 3-14 days).

After adjustment for patient characteristics, the AOR of interhospital transfer for patients without insurance compared with commercial insurance was 0.56 (95% CI, 0.51-0.61; *P* < .001) (Figure), and the estimated probability of interhospital transfer for patients without insurance was 3.20% compared with 5.59% for patients with commercial insurance (absolute difference, 2.39 percentage points). The odds of interhospital transfer were also significantly lower for patients with Medicare (AOR, 0.72; 95% CI, 0.68-0.76; *P* < .001) or Medicaid (AOR, 0.69; 95% CI, 0.64-0.74; *P* < .001) compared with patients with commercial insurance. Patients without insurance had significantly higher odds of mortality compared with patients with commercial insurance (AOR, 1.31; 95% CI, 1.25-1.37; *P* < .001) (Table 2), and the estimated probability of mortality among patients without insurance was 42.36% compared with 37.00% for patients with commercial insurance. Patients with Medicare or Medicaid did not have significant differences in odds of mortality compared with patients with commercial insurance (Medicare: AOR, 1.01 [95% CI, 0.99-1.03]; *P* = .57; Medicaid: AOR, 1.02 [95% CI, 0.99-1.05]; *P* = .20).

**Figure.**
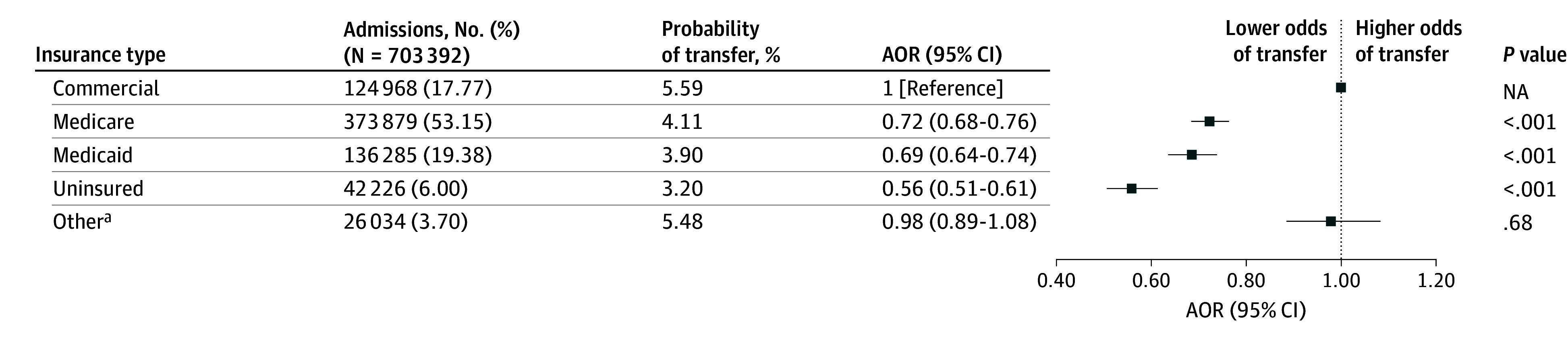
Odds of Interhospital Transfer by Patient Health Insurance Multivariable logistic regression was used to adjust for patient age, sex, comorbidities, severity of illness, and year. Cluster robust SE estimates were computed to account for hospital-level clustering. AOR indicates adjusted odds ratio; NA, not applicable. ^a^Other insurance included workers’ compensation, direct employer contracts, US Department of Defense military benefits, Indian Health Services, prison contracts, and Veterans Health Administration.

**Table 2.** Odds of Mortality by Patient Health Insurance Among Critically Ill Patients With Acute Respiratory Failure Receiving Invasive Mechanical Ventilation^a^

Patient insurance	Admissions, No. (%) (N = 703 392)	AOR (95% CI)^b^	*P* value	Estimated probability, %
Commercial	124 968 (17.77)	1 [Reference]	NA	37.00
Medicare	373 879 (53.15)	1.01 (0.99-1.03)	.57	37.10
Medicaid	136 285 (19.38)	1.02 (0.99-1.05)	.20	37.30
Uninsured	42 226 (6.00)	1.31 (1.25-1.37)	<.001	42.36
Other^c^	26 034 (3.70)	1.09 (1.02-1.16)	.007	38.62

Abbreviations: AOR, adjusted odds ratio; NA, not applicable.

^a^ Includes patients who died during admission or were discharged to hospice.

^b^ Multivariable logistic regression analyses were adjusted for patient age, sex, chronic comorbidities, severity of illness, and year of admission with cluster robust SE estimates to account for hospital-level clustering of patients.

^c^ Included workers’ compensation, direct employer contracts, US Department of Defense military benefits, Indian Health Services, prison contracts, and Veterans Health Administration.

In the model examining time to interhospital transfer, having no insurance was associated with slower time to interhospital transfer compared with having commercial insurance (AHR, 0.72; 95% CI, 0.68-0.76; *P* < .001) (Table 3). Patients with Medicare and Medicaid similarly experienced slower time to interhospital transfer compared with patients with commercial insurance (Medicare: AHR, 0.75 [95% CI, 0.72-0.77]; *P* < .001; Medicaid: AHR, 0.61 [95% CI, 0.59-0.64]; *P* < .001). Results were similar in the Fine-Gray subdistribution hazard model that accounted for censoring due to death or discharge to hospice prior to transfer (eTable 4 in Supplement 1).

**Table 3.** Associations With Slower Time to Interhospital Transfer by Patient Health Insurance Among Critically Ill Patients With Acute Respiratory Failure Receiving Invasive Mechanical Ventilation^a^

Patient insurance	No. (%)	AHR (95% CI)^b^	*P* value
Commercial	124 968 (17.77)	1 [Reference]	NA
Medicare	373 879 (53.15)	0.75 (0.72-0.77)	<.001
Medicaid	136 285 (19.38)	0.61 (0.59-0.64)	<.001
Uninsured	42 226 (6.00)	0.72 (0.68-0.76)	<.001
Other^c^	26 034 (3.70)	1.01 (0.95-1.06)	.83

Abbreviations: AHR, adjusted hazard ratio; NA, not applicable.

^a^ Includes patients who died during admission or were discharged to hospice.

^b^ The shared frailty model used a Weibull distribution and was adjusted for patient age, sex, chronic comorbidities, severity of illness, and year of admission, with clustering by hospital to account for hospital-level differences.

^c^ Included workers’ compensation, direct employer contracts, US Department of Defense military benefits, Indian Health Services, prison contracts, and Veterans Health Administration.

In subanalyses, results were similar when excluding patients who died within 7 days of admission, when excluding patient admissions to hospitals that did not transfer any patients, when excluding patients who had been transferred in from another acute care hospital, and when comparing odds of transfer stratified by hospital size and volume (eTables 5-9 in Supplement 1). Trends did not change when examining interhospital transfers stratified by the COVID-19 period (March 2020 to September 2021) (eTable 10 in Supplement 1). We observed similar patterns when stratifying analyses by patient race, ethnicity, or severity of illness (eTables 11 and 12 in Supplement 1). Patients without insurance had lower odds of interhospital transfer than patients with Medicare or Medicaid (eTable 13 in Supplement 1). Results were similar in the Fine-Gray subdistribution hazard model assessing mortality that accounted for censoring due to transfer (eTable 14 in Supplement 1).

## Discussion

Patient health insurance was associated with interhospital transfer and mortality for critically ill patients with acute respiratory failure receiving invasive mechanical ventilation. Uninsured patients and those with Medicare or Medicaid experienced decreased odds of interhospital transfer compared with patients with commercial insurance. These results were robust to analyses that accounted for the COVID-19 pandemic and hospital-level transfer practices or when stratified by severity of illness, race, or ethnicity. Patients without commercial insurance experienced slower time to interhospital transfer, even when accounting for patients who died prior to transfer. Uninsured patients also experienced increased odds of mortality compared with patients with commercial insurance.

Poor outcomes among uninsured patients with critical illness have been previously observed, even after accounting for other patient characteristics and hospital-level effects.^12,13^ The observed associations between uninsured status, lower odds of interhospital transfer, slower time to transfer, and higher odds of mortality underscore the need to explore potential health inequities in the interhospital transfer process.

A recent scoping review that evaluated interhospital transfers for acute respiratory failure emphasized the lack of evidence guiding transfer decision-making.^32^ Clinicians attempting to transfer patients to other hospitals have described barriers, such as a lack of guidance to identify patients who should be transferred and to where they should be transferred.^6^ Clinicians receiving transferred patients have reported uncertainty about which patients to prioritize for acceptance.^8^ Additionally, the extent to which patient and family preferences also might play a role in the decision for interhospital transfer is incompletely understood, with existing evidence signaling a lack of patient and family involvement in the process.^33^ Moreover, between transfer referral and transfer acceptance, patients may undergo insurance review, although limited empirical evidence is available to describe this process. This insurance review may disenfranchise patients without insurance, and perhaps, based on this study, also patients with Medicare or Medicaid insurance. Given the uncertainty surrounding interhospital transfer processes, there is potential for bias or inconsistency in clinical practices that may adversely impact socioeconomically vulnerable patients.

Several studies have similarly demonstrated an association between insurance status and interhospital transfer in conditions such as acute coronary syndrome, general medical problems, and respiratory failure.^7,10,34^ A prior study investigating potential factors affecting transfer among patients with respiratory failure similarly identified that patients with Medicare and Medicaid insurance experienced lower odds of transfer compared with patients with commercial insurance.^7^ Our study, when placed in the context of these other studies, raises questions about the role of insurance in the care of the critically ill. Conventional wisdom suggests that in the US, insurance status should play a minimal role in inpatient care. Interhospital transfer represents a relatively unique situation where insurance may be considered for advancement in care after hospitalization.

Additional work is needed to (1) evaluate whether disparities in interhospital transfer by insurance status may be related to limited reimbursement or biases toward specific groups of patients (eg, individuals with lower socioeconomic status or older patients); (2) pinpoint the rate-limiting step to interhospital transfer, whether at the referral or accepting hospital; and (3) improve understanding of which types of patients benefit from transfer. To better evaluate how insurance plays a role in the transfer process, data sources that capture more granular transfer data are crucial. For example, being able to identify patients for whom transfer was requested (not just those who were transferred) as well as reasons for requested transfer would enable a better assessment of outcomes of transferred patients and identification of potential targets for interventions to reduce disparities. Additionally, prospective data collection may be helpful to understand hospital-level processes and decision-making factors that are challenging to measure using secondary data. There is a clear need to reevaluate existing processes of care to ensure that critical care is delivered equitably.

If these disparities are related to reimbursement, a potential solution could be found from the field of emergency care. Our findings differ from studies that examined transfer patterns for patients in the emergency department prior to hospital admission. These studies showed increased odds of interhospital transfer for patients without insurance.^35,36,37,38,39^ EMTALA regulations, which mandate delivery of stabilizing care to all patients regardless of their ability to pay for care, were created to protect patients seen in hospitals for emergency care and ensure that appropriate prehospital transfers to higher levels of care occur. However, these regulations do not continue to protect patients after they are considered medically stabilized, which has typically been interpreted as after admission from the emergency department to the hospital.^40^ After medical stabilization, if hospital transfer is requested, the accepting hospital is permitted to request information about a patient’s insurance status.^40^ Given this, critically ill patients for whom interhospital transfer is requested but who may have limitations in their (or their insurer’s) ability to pay for care may be less likely to be accepted for transfer. It is possible that accepting hospitals may have a financial motivation to decline transfers of patients without insurance or with insurance with lower reimbursement. Future studies with data sources containing more granular information about transfer requests, acceptance, and denial could provide insight into this question.

If transfer denial based on insurance status is identified, multiple policy-level interventions could be considered. From a regulatory standpoint, EMTALA could be expanded to apply routinely to patients in the inpatient setting (ie, moving away from language focused on stability and toward the need for higher levels of care). The Centers for Medicare & Medicaid Services could also issue a new condition of participation preventing accepting hospitals from requesting information about ability to pay prior to transfer. To offset the financial burden of poorly reimbursed care to hospitals, federal and state reimbursement of care for transferred patients could be increased. However, it should be acknowledged that during the COVID-19 pandemic, changes were made to federal reimbursement policies to improve compensation to hospitals for providing care to patients without insurance, yet we found that the odds of interhospital transfer for patients without insurance remained lower than for patients with commercial insurance during the COVID-19 pandemic.^29,30,31^ In addition, it is possible that patients with commercial insurance may have established primary or specialty outpatient care in health systems and may be more likely to undergo transfer for continuity of care. Further assessment of the role of health insurance, as well as other nonclinical factors associated with interhospital transfers, is necessary to optimize regional critical care delivery, improve patient outcomes, and promote equitable care delivery for all patients.

### Strengths and Limitations

Our study has strengths, including being among the first to examine the association between insurance type and interhospital transfer for critically ill patients with acute respiratory failure. We used a large, nationwide sample that included a diverse sample of patients across ages and payers and accounted for factors that may impact transfer decision-making, including patient age, sex, chronic comorbidities, and severity of illness. However, our study should be interpreted in the context of several limitations. First, data were obtained from an administrative data source, and we were unable to account for all patient, clinician, and hospital factors that may impact interhospital transfer decision-making or to capture specific reasons for transfer. For example, we were unable to determine if a patient’s illness severity at the time of transfer precluded transfer, if patient or family preferences influenced transfer decisions, or if receiving-hospital capacity constraints impacted transfer acceptance. Second, we chose to focus on patients with acute respiratory failure receiving invasive mechanical ventilation using diagnosis and procedure codes, but this may inadequately capture all patients with respiratory failure. Third, we identified transferred patients based on discharge destination codes and could not follow patients across multiple hospitals or determine if patients died after transfer. Fourth, among patients who underwent interhospital transfer, we were unable to evaluate the appropriateness of transfer. Fifth, we could identify only patients who had been successfully transferred and were unable to identify patients for whom transfer was requested but ultimately declined. As a result, our findings may underestimate the true role of insurance in interhospital transfers of critically ill patients.

## Conclusions

In this cohort study, lack of health insurance was associated with lower odds of interhospital transfer and higher odds of mortality among critically ill patients. Our results signal that health insurance may play an important yet underrecognized role in the care of critically ill patients. There is a need to examine the role of insurance in the care of the critically ill and better define how interhospital transfers occur to ensure that interhospital transfer practices occur equitably.

## Notes

Supplement 1. **eFigure.** Study Cohort Diagram **eTable 1.** *ICD-10* Diagnosis and Procedure Codes Used to Define Acute Respiratory Failure and Invasive Mechanical Ventilation **eTable 2.** Proportion of Patients Cared for by Insurance Type Across Hospitals by Transfer Volume **eTable 3.** Hospital Characteristics **eTable 4.** Association Between Patient Health Insurance and Interhospital Transfer Using a Fine-Gray Subdistribution Hazard Model With Mortality as a Competing Risk **eTable 5.** Interhospital Transfer by Patient Health Insurance, Excluding Patients Who Died Within the First 7 Days of Admission **eTable 6.** Interhospital Transfer by Patient Health Insurance Among Hospitals That Transferred at Least 1 Patient (N=812 Hospitals) **eTable 7.** Interhospital Transfer by Patient Health Insurance, Excluding Patients Admitted as Interhospital Transfers **eTable 8.** Interhospital Transfer by Patient Health Insurance, Stratified by Small (<500 Beds) or Large (>500 Beds) Hospital Size **eTable 9.** Interhospital Transfer by Patient Health Insurance, Stratified by Hospital Volume **eTable 10.** Interhospital Transfer by Patient Health Insurance, Stratified by Before and During the COVID-19 Pandemic **eTable 11.** Interhospital Transfer by Patient Health Insurance, Stratified by Patient Race or Ethnicity **eTable 12.** Interhospital Transfer by Patient Health Insurance, Stratified by Patient Severity of Illness at the Time of Admission **eTable 13.** Interhospital Transfer by Patient Health Insurance Using Uninsured Status as the Reference Group **eTable 14.** Association Between Patient Health Insurance and Mortality Using a Fine-Gray Subdistribution Hazard Model With Transfer as a Competing Risk

Supplement 2.
Data Sharing Statement
