# Supplementary material for: Health Insurance and Interhospital Transfer for Critically Ill Patients With Respiratory Failure
Source: JAMA Netw Open. 2025 Aug 26;8(8):e2528889. doi: 10.1001/jamanetworkopen.2025.28889 (PMC12381673; doi:10.1001/jamanetworkopen.2025.28889)
Supplement: Supplement 1. — eFigure. Study Cohort Diagram eTable 1. ICD-10 Diagnosis and Procedure Codes Used to Define Acute Respiratory Failure and Invasive Mechanical Ventilation eTable 2. Proportion of Patients Cared for by Insurance Type Across Hospitals by Transfer Volume eTable 3. Hospital Characteristics eTable 4. Association Between Patient Health Insurance and Interhospital Transfer Using a Fine-Gray Subdistribution Hazard Model With Mortality as a Competing Risk eTable 5. Interhospital Transfer by Patient Health Insurance, Excluding Patients Who Died Within the First 7 Days of Admission eTable 6. Interhospital Transfer by Patient Health Insurance Among Hospitals That Transferred at Least 1 Patient (N=812 Hospitals) eTable 7. Interhospital Transfer by Patient Health Insurance, Excluding Patients Admitted as Interhospital Transfers eTable 8. Interhospital Transfer by Patient Health Insurance, Stratified by Small (<500 Beds) or Large (>500 Beds) Hospital Size eTable 9. Interhospital Transfer by Patient Health Insurance, Stratified by Hospital Volume eTable 10. Interhospital Transfer by Patient Health Insurance, Stratified by Before and During the COVID-19 Pandemic eTable 11. Interhospital Transfer by Patient Health Insurance, Stratified by Patient Race or Ethnicity eTable 12. Interhospital Transfer by Patient Health Insurance, Stratified by Patient Severity of Illness at the Time of Admission eTable 13. Interhospital Transfer by Patient Health Insurance Using Uninsured Status as the Reference Group eTable 14. Association Between Patient Health Insurance and Mortality Using a Fine-Gray Subdistribution Hazard Model With Transfer as a Competing Risk [file jamanetwopen-e2528889-s001.pdf]

Supplementary Online Content

Harlan EA, Ghous M, Cortinas N, et al. Health insurance and interhospital transfer for critically ill patients with respiratory failure. *JAMA Netw Open*. 2025;8(8):e2528889. doi:10.1001/jamanetworkopen.2025.28889

eFigure 1. Study Cohort Diagram
eTable 1. *ICD-10* Diagnosis and Procedure Codes Used to Define Acute Respiratory Failure and Invasive Mechanical Ventilation
eTable 2. Proportion of Patients Cared for by Insurance Type Across Hospitals by Transfer Volume
eTable 3. Hospital Characteristics
eTable 4. Association Between Patient Health Insurance and Interhospital Transfer Using a Fine-Gray Subdistribution Hazard Model With Mortality as a Competing Risk
eTable 5. Interhospital Transfer by Patient Health Insurance, Excluding Patients Who Died Within the First 7 Days of Admission
eTable 6. Interhospital Transfer by Patient Health Insurance Among Hospitals That Transferred at Least 1 Patient (N = 812 Hospitals)
eTable 7. Interhospital Transfer by Patient Health Insurance, Excluding Patients Admitted as Interhospital Transfers
eTable 8. Interhospital Transfer by Patient Health Insurance, Stratified by Small (<500 Beds) or Large (>500 Beds) Hospital Size
eTable 9. Interhospital Transfer by Patient Health Insurance, Stratified by Hospital Volume
eTable 10. Interhospital Transfer by Patient Health Insurance, Stratified by Before and During the COVID-19 Pandemic
eTable 11. Interhospital Transfer by Patient Health Insurance, Stratified by Patient Race or Ethnicity
eTable 12. Interhospital Transfer by Patient Health Insurance, Stratified by Patient Severity of Illness at the Time of Admission
eTable 13. Interhospital Transfer by Patient Health Insurance Using Uninsured Status as the Reference Group
eTable 14. Association Between Patient Health Insurance and Mortality Using a Fine-Gray Subdistribution Hazard Model With Transfer as a Competing Risk

This supplementary material has been provided by the authors to give readers additional information about their work.

© 2025 Harlan EA, et al. *JAMA Network Open*.

eFigure 1. Study Cohort Diagram

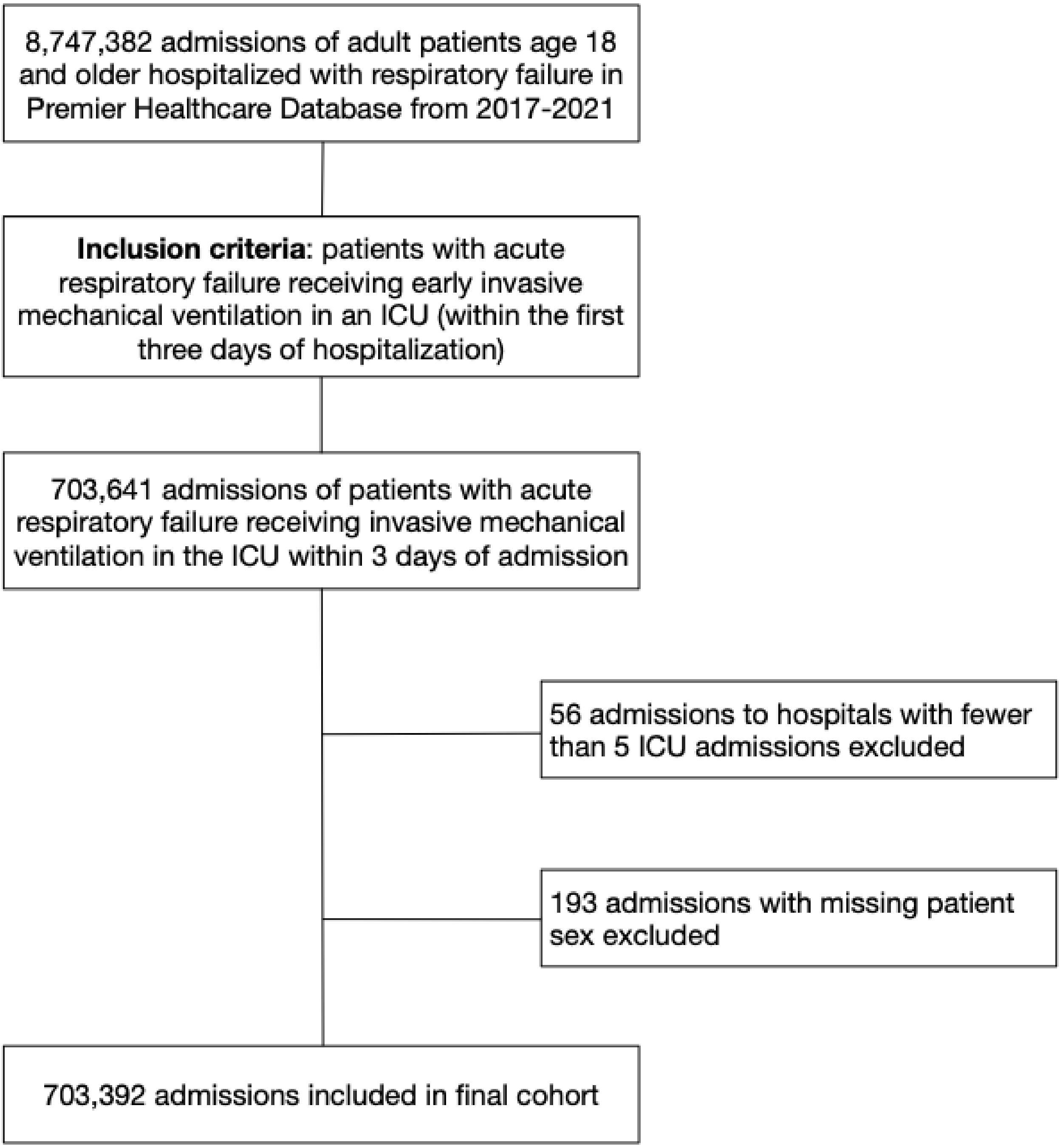

© 2025 Harlan EA, et al. *JAMA Network Open*.

eTable 1. ICD-10 diagnosis and procedure codes used to define acute respiratory failure and invasive mechanical ventilation.

	ICD-10 Code	Description
**ICD-10-CM***	J80	Acute respiratory distress syndrome
	J96.00, J96.01, J96.02	Acute respiratory failure
	J96.90, J96.91, J96.92	Respiratory failure
**ICD-10-PCS****	5A1935Z, 5A1945Z, 5A1955Z	Invasive mechanical ventilation

*International Classification of Diseases, Tenth Revision, Clinical Modification
*International Classification of Diseases, Tenth Revision, Procedure Coding System

© 2025 Harlan EA, et al. *JAMA Network Open*.

eTable 2. Proportion of patients cared for by insurance type across hospitals by transfer volume.

	**Quartile of hospital transfer volume***			
**Insurance Type**	**1 (Fewest transfers)****	**2**	**3**	**4 (Most transfers)**
**Commercial**	7,424 (18.3)	27,220 (18.2)	34,596 (17.7)	55,628 (17.5)
**Medicaid**	7,370 (18.2)	26,667 (17.8)	37,841 (19.4)	64,261 (20.2)
**Medicare**	21,969 (54.2)	80,501 (53.9)	104,490 (53.6)	166,626 (52.4)
**Uninsured**	2,526 (6.2)	9,712 (6.5)	10.932 (5.6)	19,022 (6.0)
**Other**	1,262 (3.1)	5,384 (3.6)	7,099 (3.6)	12,277 (3.9)

*Includes hospitals that transferred at least one patient (n=812)
**n (%)

© 2025 Harlan EA, et al. *JAMA Network Open*.

eTable 3. Hospital Characteristics*

	Quartile of hospital transfer volume			
	1 (Fewest transfers out)	2	3	4 (Most transfers out)
**Hospital beds****				
<200	139 (66.2)	91 (43.5)	77 (40.3)	52 (25.7)
200-400	46 (21.9)	72 (34.4)	67 (35.1)	85 (42.1)
>=400	25 (11.9)	46 (22.0)	47 (24.6)	65 (32.2)
**Rural**	73 (34.8)	44 (21.1)	37 (19.4)	31 (15.3)
**Teaching**	46 (21.9)	67 (32.1)	69 (36.1)	86 (42.6)
**U.S. Census Region**				
Midwest	54 (25.7)	59 (28.2)	48 (25.1)	41 (20.3)
Northeast	24 (11.4)	25 (12.0)	34 (17.8)	42 (20.8)
South	80 (38.1)	91 (43.5)	83 (43.5)	82 (40.6)
West	52 (24.8)	34 (16.3)	26 (13.6)	37 (18.3)

*Includes hospitals that transferred at least one patient, n = 812
**n(%)

© 2025 Harlan EA, et al. *JAMA Network Open*.

eTable 4. Association between patient health insurance and inter-hospital transfer using a Fine-Gray subdistribution hazard model with mortality as a competing risk*

Patient insurance	Estimated hazard ratio	95% CI	P value**
Commercial (n = 124,968)	Reference		
Medicare (n = 373,879)	0.77	0.75-0.80	P < 0.001
Medicaid (n = 136,285)	0.70	0.68-0.72	P < 0.001
Uninsured (n = 42,226)	0.61	0.58-0.65	P < 0.001
Other (n = 26,034)	0.95	0.90-1.00	P = 0.052

*Includes patients who died during admission or were discharged to hospice
**Adjusted for patient age, sex, chronic comorbidities, severity of illness, and year of admission.

© 2025 Harlan EA, et al. *JAMA Network Open*.

eTable 5. Inter-hospital transfer by patient health insurance, excluding patients who died within the first seven days of admission.

Patient insurance	aOR*	95% CI**	P value***	Predicted probability, %
Commercial (n = 99,786)	Reference			7.35
Medicare (n = 262,568)	0.73	0.69-0.77	P < 0.001	5.49
Medicaid (n = 111,872)	0.69	0.64-0.74	P < 0.001	5.17
Uninsured (n =32,067)	0.60	0.55-0.66	P < 0.001	4.57
Other (n = 20,085)	1.00	0.91-1.11	P = 0.971	7.36

*Adjusted odds ratio
**Confidence interval
***Analyses are adjusted for patient age, sex, chronic comorbidities, severity of illness, and year of admission with cluster robust standard error estimates to account for hospital-level clustering of patients.

© 2025 Harlan EA, et al. *JAMA Network Open*.

eTable 6. Inter-hospital transfer by patient health insurance among hospitals that transferred at least one patient (n = 812 hospitals).

Patient insurance	aOR*	95% CI**	P value***	Predicted probability, %
Commercial (n = 124,868)	Reference			5.59
Medicare (n = 373,586)	0.72	0.68 – 0.76	P < 0.001	4.11
Medicaid (n = 136,139)	0.69	0.64 – 0.74	P < 0.001	3.91
Uninsured (n = 42,192)	0.56	0.51 – 0.61	P < 0.001	3.20
Other (n = 26,022)	0.98	0.89 – 1.08	P = 0.678	5.48

*Adjusted odds ratio
**Confidence interval
***Analyses are adjusted for patient age, sex, chronic comorbidities, severity of illness, and year of admission with cluster robust standard error estimates to account for hospital-level clustering of patients.

© 2025 Harlan EA, et al. *JAMA Network Open*.

eTable 7. Inter-hospital transfer by patient health insurance, excluding patients admitted as inter-hospital transfers.

Patient insurance	aOR	95% CI	P value	Predicted probability, %
Commercial (n = 101,824)	Reference			5.95
Medicare (n = 311,292)	0.73	0.68-0.77	P < 0.001	4.39
Medicaid (n = 112,908)	0.65	0.61 – 0.71	P < 0.001	3.98
Uninsured (n = 35,848)	0.50	0.45 – 0.56	P < 0.001	3.09
Other (n = 21,182)	0.91	0.82 – 1.01	P = 0.091	5.47

*Analyses are adjusted for patient age, sex, chronic comorbidities, severity of illness, and year of admission with cluster robust standard error estimates to account for hospital-level clustering of patients.

© 2025 Harlan EA, et al. *JAMA Network Open*.

eTable 8. Inter-hospital transfer by patient health insurance, stratified by small (< 500 beds) or large ($\geq$ 500 beds) hospital size.

Patient insurance	aOR	95% CI	P value*	Predicted probability, %
**Commercial**				
Small hospitals (n = 69,652)	Reference			7.76
Large hospitals (n = 55,316)				2.79
**Medicare**				
Small hospitals (n = 221,992)	0.71	0.68-0.76	P < 0.001	5.68
Large hospitals (n = 151,887)	0.66	0.58-0.75	P < 0.001	1.86
**Medicaid**				
Small hospitals (n = 78,261)	0.66	0.62-0.71	P < 0.001	5.28
Large hospitals (n = 58,024)	0.69	0.57-0.84	P < 0.001	1.94
**Uninsured**				
Small hospitals (n = 22,760)	0.55	0.50-0.61	P < 0.001	4.42
Large hospitals (n = 19,466)	0.60	0.48-0.75	P < 0.001	1.69
**Other**				
Small hospitals (n = 14,722)	0.87	0.78-0.96	P = 0.006	6.79
Large hospitals (n = 11,312)	1.36	1.10-1.70	P = 0.005	3.77

*P values compare patients from each hospital group to patients with commercial insurance from the same hospital group. Analyses are adjusted for patient age, sex, chronic comorbidities, severity of illness, and year of admission with cluster robust standard error estimates to account for hospital-level clustering of patients.

© 2025 Harlan EA, et al. *JAMA Network Open*.

eTable 9. Inter-hospital transfer by patient health insurance, stratified by hospital volume.

Hospital Volume	aOR	95% CI	P value*	Predicted probability, %
**Quartile 1, Lowest Volume (n = 11,615, 1.65%)**				
Commercial (n = 1,983)	Reference			22.60
Medicare (n = 6,305)	0.92	0.78-1.07	P=0.001	21.14
Medicaid (n = 2,344)	0.76	0.64-0.89	P=0.120	18.22
Uninsured (n = 622)	0.82	0.64-1.05	P=0.274	19.38
Other (n = 361)	0.88	0.66-1.16	P=0.356	20.43
**Quartile 2 (n = 61,481, 8.74%)**				
Commercial (n = 10,553)	Reference			12.66
Medicare (n = 34,236)	0.77	0.69-0.85	P < 0.001	10.02
Medicaid (n = 11,363)	0.70	0.63-0.78	P < 0.001	9.25
Uninsured (n = 3,284)	0.70	0.61-0.79	P < 0.001	9.19
Other (n =2,045)	0.97	0.81-1.17	P = 0.754	12.34
**Quartile 3 (n = 156,820, 22.29%)**				
Commercial (n = 26,218)	Reference			8.44
Medicare (n = 88,806)	0.68	0.63-0.75	P < 0.001	6.00
Medicaid (n = 29,042)	0.65	0.59-0.72	P < 0.001	5.66
Uninsured (n = 7,611)	0.47	0.40-0.54	P < 0.001	4.14
Other (n = 5,143)	0.87	0.76-0.99	P = 0.043	7.43
**Quartile 4, Highest volume (n = 473,476, 67.31%)**				
Commercial (n = 86,214)	Reference			3.38
Medicare (n = 244,532)	0.65	0.60-0.70	P < 0.001	2.22
Medicaid (n = 93,536)	0.65	0.58-0.73	P < 0.001	2.24
Uninsured (n = 30,709)	0.56	0.48-0.65	P < 0.001	1.92
Other (n = 18,485)	1.14	0.98-1.32	P = 0.079	3.83

*Analyses are adjusted for patient age, sex, chronic comorbidities, severity of illness, and year of admission with cluster robust standard error estimates to account for hospital-level clustering of patients.

© 2025 Harlan EA, et al. *JAMA Network Open*.

eTable 10. Inter-hospital transfer by patient health insurance, stratified by before and during the COVID-19 pandemic.

Patient insurance	aOR	95% CI	P value*	Predicted probability, %
**Commercial**				
Pre-COVID (n = 84,393)	Reference			5.77
COVID era (n = 40,575)				5.21
**Medicare**				
Pre-COVID (n = 259,104)	0.72	0.67 – 0.76	P < 0.001	4.21
COVID (n = 114,775)	0.73	0.68 – 0.79	P < 0.001	3.88
**Medicaid**				
Pre-COVID (n = 89,956)	0.69	0.63 – 0.74	P < 0.001	4.03
COVID (n = 46,329)	0.68	0.63 – 0.75	P < 0.001	3.63
**Uninsured**				
Pre-COVID (n = 30,017)	0.57	0.52 – 0.63	P < 0.001	3.39
COVID (n = 12,209)	0.51	0.44 – 0.60	P < 0.001	2.76
**Other**				
Pre-COVID (n = 16,946)	1.00	0.89 – 1.11	P = 0.974	5.76
COVID (n = 9,088)	0.95	0.82 – 1.09	P = 0.439	4.95

*P values compare patients from each time period to patients with commercial insurance from the same time period. Analyses are adjusted for patient age, sex, chronic comorbidities, severity of illness, and year of admission with cluster robust standard error estimates to account for hospital-level clustering of patients.

© 2025 Harlan EA, et al. *JAMA Network Open*.

eTable 11. Inter-hospital transfer by patient health insurance, stratified by patient race or ethnicity.

Race or ethnicity	aOR	95% CI	P value*	Predicted probability, %
**Asian (n = 15,432, 2.19%)**				
Commercial (n = 3,439)	Reference			6.42
Medicare (n = 8,058)	0.71	0.51 – 0.98	P = 0.04	4.63
Medicaid (n = 2,891)	0.57	0.42 – 0.78	P < 0.001	3.79
Uninsured (n =599)	0.56	0.36 – 0.88	P = 0.011	3.72
Other (n = 445)	0.98	0.63 – 1.52	P = 0.914	6.28
**Black (n = 115,384, 16.40%)**				
Commercial (n = 16,428)	Reference			4.78
Medicare (n = 59,297)	0.74	0.67 – 0.83	P < 0.001	3.60
Medicaid (n = 28,236)	0.72	0.64 – 0.81	P < 0.001	3.50
Uninsured (n = 7,583)	0.58	0.49 – 0.69	P < 0.001	2.85
Other (n = 3,840)	1.13	0.93 – 1.37	P = 0.210	5.37
**White (n = 487,832, 69.35%)**				
Commercial (n = 90,239)	Reference			5.53
Medicare (n = 270,413)	0.75	0.71 – 0.80	P < 0.001	4.22
Medicaid (n = 82,120)	0.71	0.66 – 0.77	P < 0.001	4.02
Uninsured (n = 27,044)	0.58	0.52 – 0.65	P < 0.001	3.30
Other (n = 18,016)	1.00	0.90 – 1.11	P = 0.996	5.53
**Hispanic* (n = 59,003, 8.39%)**				
Commercial (n = 9,035)	Reference			4.88
Medicare (n = 25,688)	0.74	0.63 – 0.87	P < 0.001	3.68
Medicaid (n = 15,186)	0.79	0.65 – 0.96	P = 0.020	3.91
Uninsured (n = 6,669)	0.52	0.42 – 0.64	P < 0.001	2.58
Other (n = 2,425)	0.80	0.59 – 1.09	P = 0.163	3.97

*Ethnicity reported separately from race; patients could be included in both a race and ethnicity category

**Analyses are adjusted for patient age, sex, chronic comorbidities, severity of illness, and year of admission with cluster robust standard error estimates to account for hospital-level clustering of patients.

© 2025 Harlan EA, et al. *JAMA Network Open*.

eTable 12. Inter-hospital transfer by patient health insurance, stratified by patient severity of illness at the time of admission.

Patient insurance	aOR	95% CI	P value*	Predicted probability, %
**0-1 Organ Failures (n = 444,185 , 63.15%)**				
Commercial (n = 82,424)	Reference			5.55
Medicare (n =227,772)	0.75	0.71 – 0.81	P < 0.001	4.25
Medicaid (n = 88,259)	0.70	0.64 – 0.76	P < 0.001	3.96
Uninsured (n =28,249)	0.55	0.50 – 0.62	P < 0.001	3.16
Other (n = 17,481)	1.01	0.91 – 1.13	P = 0.820	5.62
**2-3 Organ Failures (n = 232,847, 33.10%)**				
Commercial (n = 37,518)	Reference			5.86
Medicare (n = 133,674)	0.66	0.62 – 0.71	P < 0.001	3.98
Medicaid (n = 42,071)	0.65	0.60 – 0.71	P < 0.001	3.92
Uninsured (n = 11,940)	0.58	0.51 – 0.65	P < 0.001	3.47
Other (n = 7,644)	0.91	0.80 – 1.05	P = 0.190	5.39
**4 or More Organ Failures (n = 26,360, 3.75%)**				
Commercial (n = 5,026)	Reference			4.39
Medicare (n = 12,433)	0.66	0.54 – 0.81	P < 0.001	2.94
Medicaid (n = 5,955)	0.55	0.45 – 0.68	P < 0.001	2.48
Uninsured (n = 2.037)	0.40	0.29 – 0.56	P < 0.001	1.82
Other (n = 909)	0.84	0.59 – 1.20	P = 0.343	3.72

*Analyses are adjusted for patient age, sex, chronic comorbidities, and year of admission with cluster robust standard error estimates to account for hospital-level clustering of patients. Organ failures excludes respiratory failure.

© 2025 Harlan EA, et al. *JAMA Network Open*.

eTable 13. Inter-hospital transfer by patient health insurance using uninsured status as the reference group.

Patient insurance	aOR*	95% CI**	P value***	Predicted probability, %
Uninsured (n = 42,226)	Reference			3.20
Commercial (n = 124,968)	1.80	1.63-1.98	P < 0.001	5.59
Medicare (n = 373,879)	1.30	1.18-1.42	P < 0.001	4.11
Medicaid (n = 136,285)	1.23	1.10-1.37	P < 0.001	3.90
Other (n = 26,034)	1.76	1.56-1.98	P < 0.001	5.48

*Adjusted odds ratio
**Confidence interval
***Analyses are adjusted for patient age, sex, chronic comorbidities, severity of illness, and year of admission with cluster robust standard error estimates to account for hospital-level clustering of patients. Organ failures excludes respiratory failure.

© 2025 Harlan EA, et al. *JAMA Network Open*.

eTable 14. Association between patient health insurance and mortality using a Fine-Gray subdistribution hazard model with transfer as a competing risk.*

Patient insurance	Estimated hazard ratio	95% CI	P value**
Commercial (n = 124,968)	Reference		
Medicare (n = 373,879)	1.11	1.10-1.13	P < 0.001
Medicaid (n = 136,285)	1.02	1.01-1.04	P = 0.004
Uninsured (n = 42,226)	1.39	1.36-1.42	P < 0.001
Other (n = 26,034)	1.07	1.04-1.09	P < 0.001

*Includes patients who died during admission or were discharged to hospice

**Adjusted for patient age, sex, chronic comorbidities, severity of illness, and year of admission.

© 2025 Harlan EA, et al. *JAMA Network Open*.
