# Supplementary material for: Health Insurance and Interhospital Transfer for Critically Ill Patients With Respiratory Failure
Source: JAMA Netw Open. 2025 Aug 26;8(8):e2528889. doi: 10.1001/jamanetworkopen.2025.28889 (PMC12381673; doi:10.1001/jamanetworkopen.2025.28889)
Supplement: Supplement 2. — Data Sharing Statement [file jamanetwopen-e2528889-s002.pdf]

Data Sharing Statement

Harlan. Health Insurance and Interhospital Transfer for Critically Ill Patients With Respiratory Failure. *JAMA Netw Open*. Published August 26, 2025.
doi:10.1001/jamanetworkopen.2025.28889

Data
Data available: No

Additional Information
Explanation for why data not available: The data in this study are proprietary to the Premier Healthcare Database and cannot be shared freely. However, the statistical code used in this study is available on request to the corresponding author.
